# Novel molecular diagnostic tools for malaria elimination: a review of options from the point of view of high-throughput and applicability in resource limited settings

**DOI:** 10.1186/s12936-016-1158-0

**Published:** 2016-02-16

**Authors:** Sumudu Britton, Qin Cheng, James S. McCarthy

**Affiliations:** University of Queensland, Brisbane, Australia; QIMR Berghofer Medical Research Institute, Brisbane, Australia; Australian Army Malaria Institute, Brisbane, Australia

**Keywords:** Malaria elimination, High-throughput, Molecular diagnostics, LAMP, PCR, Field application

## Abstract

As malaria transmission continues to decrease, an increasing number of countries will enter pre-elimination and elimination. To interrupt transmission, changes in control strategies are likely to require more accurate identification of all carriers of *Plasmodium* parasites, both symptomatic and asymptomatic, using diagnostic tools that are highly sensitive, high throughput and with fast turnaround times preferably performed in local health service settings. Currently available immunochromatographic lateral flow rapid diagnostic tests and field microscopy are unlikely to consistently detect infections at parasite densities less than 100 parasites/µL making them insufficiently sensitive for detecting all carriers. Molecular diagnostic platforms, such as PCR and LAMP, are currently available in reference laboratories, but at a cost both financially and in turnaround time. This review describes the recent progress in developing molecular diagnostic tools in terms of their capacity for high throughput and potential for performance in non-reference laboratories for malaria elimination.

## Background

Malaria continues to be a significant public health issue, with 198 million cases of infection occurring in 2013 [1]. However, it is estimated that 55 of the 106 countries that reported ongoing transmission in the year 2000 will, by 2015, meet the target of reducing malaria case incidence rates by 75 %. In this context, in 2007, the World Health Organization (WHO) endorsed the ambitious goal of achieving worldwide malaria elimination and eradication. The definitions and associated challenges of elimination have been previously discussed [2, 3].

Inherent to a change in focus from control to elimination is the need to interrupt transmission, which requires identification and treatment of all parasite carriers, both symptomatic and asymptomatic. To this end, there is increasing appreciation of the extent of the asymptomatic reservoir of malaria parasites, their potential to maintain transmission and the role that sensitive diagnostic tests will need to play in providing accurate epidemiological information to guide programmatic changes. A review by Okell et al. found that for *Plasmodium falciparum,* microscopy underestimated prevalence by 50.8 % compared with PCR, and that this gap became even more significant in low transmission settings [4]. Similarly, Cheng et al. described submicroscopic *Plasmodium vivax* infection being highly prevalent particularly in areas of low transmission, with on average 69.5 % of infections being detected only by PCR [5].

The diagnostic tools currently available for the identification of *Plasmodium* spp. include light microscopy, immunochromatographic lateral flow assays (known as rapid diagnostic tests, RDTs), serology, fluorescence microscopy and nucleic acid amplification techniques (NATs), such as PCR and isothermal amplification. Light microscopy has poor sensitivity in low transmission setting and in asymptomatic patients, resulting in underestimation of disease prevalence compared with the gold standard molecular diagnostic tool, PCR [4–6]. Even the most sensitive RDTs share similar limitations with microscopy [7], and have been shown to be insufficiently sensitive for community screening of asymptomatic carriers of *P. falciparum* [8, 9]. Furthermore, mass screening of populations in five malaria ‘hot spots’ in Zanzibar and treatment of RDT positive individuals did not reduce malaria incidence [9], likely due to the nearly tenfold higher undetected and untreated infection reservoirs missed by RDTs (compared with PCR). Therefore, diagnostic tools with better analytical sensitivity are likely to be required to detect the low level parasitaemia associated with asymptomatic carriage of *Plasmodium* spp. pertaining to the goal of malaria elimination.

The Malaria Eradication consortium has outlined features required of diagnostic tools for the purpose of malaria elimination [10]. These were further modified by the WHO in 2014 [11] to include a lower limit of two parasites/µL detected in order to be ‘a significant improvement on expert microscopy’. However, the minimum number of parasites per microlitre that perpetuates transmission in low transmission settings remains uncertain.

For malaria elimination, the strategies for interrupting the cycle of transmission rely on enhanced population surveillance to inform additional intervention. Surveillance for elimination includes reactive and proactive active case detection, mass screening/testing and treatment (MSAT/MTAT), focused screening and treatment (FSAT) [12] and mass drug administration (MDA) [13, 14]. However, the most effective of these strategies remains to be determined. While the success of each of these strategies will depend on a number of different factors, the feature common to each of them, except MDA, may be the availability of point-of-care, sensitive diagnostic tools that are able to process large numbers of samples with a fast turnaround time.

NATs are currently the most sensitive diagnostic modality available, and many different assays have been developed for the identification of *Plasmodium* parasites. The performance of these assays, in terms of their accuracy, has been comprehensively reviewed recently [15]. However, while readily available in reference laboratories, NATs such as PCR remains inaccessible in resource limited settings due to the expensive infrastructure, reagents and technical expertise required. Efforts to improve the field applicability of NATs have resulted in the development of assays such as PCR-enzyme linked immunosorbent assays (PCR-ELISA) [16], nucleic acid lateral flow immunoassay (NALFIA) [17, 18], nested PCR-high resolution melting analysis (nPCR-HRM) [19], PCR-ligase detection reaction assays (PCR-LDR) [20] and modifications of PCR-LDR into LDR- fluorescent microsphere assay (LDR-FMA) [21]. These assays, which have been recently reviewed [22], have good analytical sensitivity but are limited in their field applicability by virtue of their requirement for a PCR amplification step.

Isothermal molecular diagnostic modalities, such as loop mediated isothermal amplification (LAMP), nucleic acid sequence based amplification (NASBA), thermophilic helicase dependent amplification (tHDA) and recombinase polymerase amplification (RPA), which have also been detailed elsewhere [23], are of much current interest given their potential to combine good analytical sensitivity with technical requirements that may facilitate application in resource limited settings.

Several NATs, including quantitative PCR (qPCR) combined with microfluidics [24], and those linked to enzyme-linked immunosorbent assays [25–27] as well as non-nucleic acid techniques [28–30] are in development for the purposes of malaria diagnosis but are not discussed further here. However, for even the most promising molecular technologies to be useful as point of care (POC) tests, significant challenges of portability, sample preparation, power supply and high throughput [31] have yet to be overcome.

This review describes the currently available novel molecular diagnostic platforms for the detection of low parasitaemia from the point of view of their potential for high-throughput and applicability in resource limited settings for the purpose of malaria elimination.

## Nucleic acid amplification techniques (NATs)

PCR is a very sensitive molecular diagnostic modality capable of identifying asymptomatic, submicroscopic infection. The sensitivity of PCR for identifying low level parasitaemia has been shown to be improved by performing qPCR using DNA from high blood volumes (up to 1 ml). The latter platform, as described by Imwong et al., has a limit of detection (LOD) of 0.022 parasites/µL [32]. This has been achieved using a modified Qiagen© extraction protocol with extraction of DNA from 1 ml of blood, then concentrated to a volume of 10 µL of DNA with 2 µL of template per reaction (therefore equivalent to 200 µL of blood) in combination with primers to a *P. falciparum* 18S rRNA gene target [32]. The process is amenable to up-scaling through automation to test up to 700 samples per week, confirming its status as a high-throughput diagnostic modality. While this technique, when offered in a reference laboratory, could make a significant contribution to the detection of low-level parasitaemia, its significant drawback is the need for venipuncture, which is impractical for population surveillance purposes. In addition, the equipment, reagents and technical expertise required are likely to preclude its use as a field deployable test. Nevertheless, it highlights the significant diagnostic advantages associated with sample concentration for identification of low level parasitaemia. The assay has been shown capable of identifying most infected individuals in malaria-endemic areas based on the distribution of parasite densities in asymptomatic malaria determined by this assay [33].

Efforts to simplify PCR for the purpose of field application have seen PCR performed directly on blood and combined with NALFIA [18] and multiplex malaria sample ready (MMSR) assays [34]. The MMSR assay relies on the lyophilization of all multiplex qPCR reagents in either 8-tube strips or 96 well plates which requires only the addition of sample DNA and water for the identification of *Plasmodium* genus and *P. falciparum* at an LOD of 0.244 parasites/µL from whole blood. The assay can also identify *P. vivax.* Although MMSR still requires a thermocycler, DNA extraction and is expensive (> US$10 per reaction), it overcomes some significant issues associated with performing PCR in resource limited settings, thereby providing proof-of-concept for the potential PCR has to become deployable in non-reference laboratory settings.

To overcome the issue of equipment in field settings, Canier et al. created a mobile molecular laboratory [35]. At a cost of US$200,000 for the fully equipped truck, this laboratory is able to process 240 samples per day, with a turnaround time of 24 h and a cost of US$2.75/sample. The real time assay was performed in a 96-well plate using DNA extracted from filter paper using a commercial kit (Instagene Matrix resin, BioRad). Samples were screened for *Plasmodium* spp. using a mitochondrial cytochrome-b target [36] and then a species-specific nested real time PCR performed to arrive at an LOD of two parasites/µL from a 5 µL dried blood spot (DBS) [35]. While this innovative approach provides the opportunity for performing field-based PCR with its associated high throughput and fast turnaround time, the cost outlay of the mobile laboratory and cost per sample may be prohibitive in many developing countries embarking on malaria elimination programmes.

A significant advantage of real-time PCR is the lack of post-PCR handling through the use of fluorophores. To overcome issues of expense, Lucchi et al. [37] developed a novel, low cost technique for labelling real-time PCR primers with self-quenching ability via photo-induced electron transfer (PET). The PET-PCR assay was performed on Qiagen©-extracted DNA using a duplex of *Plasmodium* genus and *P. falciparum* primers, and was found to have an LOD of 3.2 parasites/µL [37]. Validation of the assay on nearly 3000 surveillance samples from Haiti, took over 5 months to process [38]. Therefore, while PET-PCR would appear to offer a sensitive platform for performing real time PCR, it is unclear whether the overall cost of the technology coupled with turnaround time would render it amenable for field deployment for malaria elimination.

Portable, self-contained molecular technology is the ultimate prize in the development of field applicable diagnostic tools. The first of these is based on a plastic hydrogel chip and a customized portable real time PCR machine (Gelcycler) [39]. The assay can process 12 samples at a time, directly from whole blood, with no prior DNA extraction. The assay uses an 18S rRNA gene target that detects all species of *Plasmodium* with an LOD of two parasites/µL and differentiated between *P. falciparum* and *P. vivax* [39]. The assay had a 2 h turnaround time using a 12 volt battery and costs US$1 per test and US$2000 for the gelcycler [39]. Although able to process only 12 samples at a time, the low cost, self contained nature of this chip and ability to test blood directly with a fast turnaround time make it an enticing prospect for field applicable diagnostics. The second platform is a multiplex microarray assay, targeting 26 tropical pathogen species including *P. falciparum* and *P. vivax,* with a turnaround time of 4 h [40]. However, its cost and loss of sensitivity at less than 100 parasites/µL for *P. falciparum* suggest a limited role for the assay for the purpose of malaria elimination.

Another approach to increasing sample throughput is pooling of samples [41]. In this study by Hsiang et al., filter paper samples were pooled into groups of 10, 50 and 100 and tested by real time PCR and species differentiated using a restriction enzyme analysis. The study found that at 10 parasites/µL, a pool of ten had a sensitivity of 93 % for *P. falciparum* [41] but turnaround time, throughput and cost of this approach are unclear. However, pooling strategies are only likely to be cost effective in areas with very low level transmissions, and where parasitaemic subjects have a moderate parasitaemia, which may permit pooling of DNA samples, thereby limiting its potential role in elimination settings.

Finally, RNA assays are generally considered too technically challenging for application in resource limited settings. Nevertheless, Cheng et al. have developed a capture and ligation probe PCR (CLIP-PCR) where sample lysate are directly incubated with a target plate overnight, obviating need for RNA purification and reverse transcription. qPCR can then be performed and results analysed by melting curve analysis and cycle threshold values. It is high throughput by virtue of its 96-well plate format and sample pooling, and can be performed directly from whole blood with an LOD of 0.05 parasites/µL or from pooled DBS with an LOD of 0.3 parasites/µL [42]. More than 3000 DBS were processed in the study but throughput per day is unclear. Limitations are an overnight incubation step which extends turnaround time, ability to identify only *Plasmodium* genus and requirement for PCR. Nevertheless, the potential for high throughput and direct processing from blood opens the possibilities for RNA assays to enter the foray of diagnostics for malaria elimination.

## Loop mediated isothermal amplification (LAMP)

Since its first description in 2001 by Mori et al. [43], LAMP has become a forerunner in isothermal diagnostic technology for the identification of *Plasmodium* species [23]. LAMP has been used to identify all human *Plasmodium* species [44] including *Plasmodium knowlesi* [45, 46], and LAMP primers have been optimized to improve the sensitivity with which *P. falciparum* [47, 48] and *P. vivax* [49, 50] can be detected. A commercially available Loopamp kit (Eiken Chemical co) has been validated for the detection of *P*. *falciparum* [51] and enables the indirect detection of *P. vivax* using a combination of pan-genus and *P. falciparum* specific LAMP primers [52]. Given that the commercial LAMP turbidimeter has limited capacity, Loopamp kits have been successfully combined with 46-well heat blocks and UV lamps to increase throughput [53, 54]. Nevertheless, further optimization of LAMP platforms will be needed in order to viably process large numbers of samples as might be required to broaden its applicability for malaria elimination [31, 55].

Goto et al. [56] described a simple LAMP platform to improve its throughput using a 96-well microtitre plate format and a metal ion detector, hydroxynaphthol blue (HNB), for rapid visual interpretation of positive and negative results. This platform has been adapted for the identification of *Plasmodium* genus and *P. falciparum* as a high-throughput LAMP (HtLAMP) assay that can be combined with a portable photospectrometer for objective confirmation of visually detectable HNB colour change results [57]. Its LOD for the identification of *Plasmodium* genus (HtLAMP-Pg) is 2.5 parasites/µL from whole blood and 25 parasites/µL from DBS. The cost of HtLAMP-Pg is US$1 per sample which includes whole blood DNA extraction using a modified chelex-saponin protocol. Although HtLAMP requires DNA extraction and is yet to be validated on asymptomatic patient samples to confirm throughput capacity, it has been performed in a resource limited setting and offers a cost effective molecular diagnostic platform.

Notwithstanding the issue of high-throughput, one of the exciting aspects of LAMP has been its potential for field deployment by virtue of its isothermal nature, and as such efforts have been invested in improving its field readiness. Lucchi et al. described the RealAmp platform in which LAMP was housed in a portable tube scanner with a built-in fluorescent detection unit able to monitor the generation of fluorophores by SYBR green in real time rather than reliance on visual colour change [58]. The device is a compact, 8 tube well, closed-system with an LOD of 0.4–40 parasites/µL for *Plasmodium* genus detection depending on method of DNA extraction. Costs are quoted at US$6344 start-up and US$2.66 per sample at minimum. RealAmp has also been used to detect *P. vivax* [49], and has been validated in field testing [59].Therefore, while this platform itself appears portable and relatively sensitive, its potential role in malaria elimination may be limited by its throughput and cost.

The concept of lateral flow based immunodiagnostics is familiar to the field of malaria. LAMP has been combined with a lateral flow device (LFD) thus adapting a molecular assay into a rapid diagnostic device [60]. This LAMP-LFD assay, performed on extracted DNA or directly on blood, targets the dihydrofolate reductase thymidylate synthase (dhfr-ts) genes of *P. falciparum* and *P. vivax* using biotynylated LAMP primers whose products then hybridized with FITC labelled single strand DNA. A strepavidin-biotin reaction between hybridized LAMP amplicons and gold-labelled anti-FITC antibodies on the LFD strip allow visualization of the result [60]. However, this LAMP-LFD platform has limited sensitivity at low-level parasitaemia, and has not been validated on clinical samples. Importantly, it is a multistep process, requiring the addition of the probe at the end of the LAMP reaction, making this open system vulnerable to contamination, particularly where high throughput sample processing is necessary. Nevertheless, the potential for simple visualization of results offered by LFD, combined with either LAMP [60], recombinase polymerase amplification (RPA) [61] or PCR [18], is promising.

Field applicability of diagnostics devices centre around simplicity of the assay and ability to be performed with minimal instrumentation but generally still depend on a reliable electricity supply. Non-instrumented nucleic acid amplification (NINA) [62] has been applied to the detection of *Plasmodium* species as a LAMP-NINA assay [63]. This assay uses an exothermic chemical reaction between saline and a magnesium iron alloy to generate a heat source for performing LAMP on a maximum of five samples within an insulated thermos flask-like device, and was validated in Ethiopia using Loopamp malaria pan genus/Pf kits (Eiken chemical co, Japan) in the NINA heating device [63]. Its limitations are its lack of capacity for high throughput, risk of contamination and validation only in samples with relatively high parasitaemia. However, the self-contained electricity free heating device, lack of post processing handling and fast turnaround time make it an attractive prospect as a field applicable point of care diagnostic device.

## Conclusion

The extent of the submicroscopic parasite reservoir is becoming more evident with the utilization of sensitive, reference laboratory-based PCR testing. Microscopy and RDTs have been shown to underestimate the *Plasmodium* parasite prevalence in low incidence settings when compared with PCR, which has important implications for malaria elimination [9, 64]. The availability of sensitive diagnostic tests would also facilitate improved diagnosis of causes of febrile illness as malaria incidence declines and allow for more appropriate administration of artemisinin combination therapy (ACT) [19]. Although many diagnostic platforms may be capable of fulfilling this role, given the sensitivity of NATs, there is an increasing priority for the development of molecular diagnostic tools that have the capacity for detection of low level parasitaemia, that are able to process large numbers of samples and that can be performed in regional, non reference laboratory settings in order to support efforts of malaria elimination.

The various novel PCR and LAMP platforms currently available (Table 1) have demonstrated variable analytical sensitivity; many of these have yet to be validated among asymptomatic patients for the specific purpose of identifying low-level parasitaemia and have yet to be evaluated in resource limited settings. The main dichotomy between the two types of molecular assays remains that PCR-based assays have the sensitivity of parasite detection and the infrastructure support to increase throughput of sample processing, but have limited capacity to be deployable to more resource-limited laboratory settings. Isothermal assays, such as LAMP on the other hand, appear to have adequate analytical sensitivity to detect low-level parasitaemia and strong potential for deployment in non-reference laboratory settings but have limited, often implied rather than proven, capacity for high throughput processing of samples. Furthermore, many of these platforms are yet to overcome the common hurdle of simplifying nucleic acid extraction, an essential requirement for such assay platforms in resource limited settings. Both PCR and LAMP based assays are currently more expensive than RDT. Nevertheless, engineering challenges may be surmountable and strong foundations have been laid for achieving the goal of developing sensitive molecular diagnostic tools with the capacity for both field deployment and high throughput sample processing for the purpose of detecting low-level parasitaemias in malaria elimination settings.

**Table 1:** Summary of capacity for high-throughput and potential for field applicability of microscopy, RDT and novel nucleic acid amplification platforms

Diagnostic modality	Platform	Limit of detection (parasites/µL)	Number of samples processed (per assay or per day)	Validated on symptomatic or microscopy positive samples	Validated on asymptomatic or surveillance samples	Assay evaluated in resource-limited setting	Cost^a^
Microscopy	Light microscopy [65]	50–499	15 min/slide^c^	Yes	Yes	Yes	$
RDT	Immunochromatographic lateral flow assay^b^ [8]	200	15–20 min/test^d^	Yes	Yes	Yes	$
PCR	Ultrasensitive qPCR [32]	0.022 (blood)	40 samples/day (manual) 140 samples/day (automated)	Yes	No	No	$$
	Multiplex Malaria Sample Ready [34]	0.244 (blood)	uncertain	Uncertain	Uncertain	Yes	$$
	Mobile PCR [35]	2 (DBS)	96 well plate, 240 samples/day	Uncertain	Yes	Yes	$$
	PET-PCR [37, 38]	3.2 (blood)	Real-time PCR platform, presumed 72 samples	Uncertain	Yes	No	?
	Gelcycler [39]	2 (blood)	12	Yes	Yes	No	$
	Multiplex microarray assay [40]	Uncertain	Uncertain	Yes	Uncertain	No	$$
	CLIP-PCR [42]	0.3 (DBS)	96 well plate + pooled DBS	Yes	Yes	No	$
LAMP	RealAmp [58]	0.4–40 (blood)	8 tubes	Yes	Yes	Yes	$$
	HtLAMP [57]	2.5 (blood)	96 well plate	Yes	No	Yes	$
	LAMP-LFD [60]	Uncertain	1.5 h/test	Yes	No	No	?
	NINA-LAMP [63]	Uncertain	5 tubes	Yes	Yes	Yes	?
	LoopAmp [51]	5 (blood)	16	Yes	Yes	Yes	?

*DBS* dried blood spot

^a^$ < $US2; $$ > $US2, ? = cost unknown

^b^Malaria Rapid Diagnostic Test Performance—results of WHO product testing of malaria RDTs Round 5 (2013)

^c^WHO Malaria Microscopy Quality Assurance Manual Version 1, 2009

^d^CDC website ww.cdc.gov/malaria/malaria_worldwide/reduction/dx_rdt.html
